# (2*E*)-*N*-(3,5-Dibromo-4-methoxy­phen­yl)-2-(hydroxy­imino)acetamide

**DOI:** 10.1107/S1600536810018623

**Published:** 2010-05-22

**Authors:** Simon J. Garden, Angelo C. Pinto, Fernanda R. da Cunha, Silvia P. Fontes, A. S. Lima, Edward R. T. Tiekink

**Affiliations:** a Instituto de Química, Departamento de Quimica Orgânica, Universidade Federal do Rio de Janeiro, Ilha do Fundão, CT, Bloco A, Rio de Janeiro 21949-900, RJ, Brazil; b Department of Chemistry, University of Malaya, 50603 Kuala Lumpur, Malaysia

## Abstract

The title compound, C_9_H_8_Br_2_N_2_O_3_, is planar (r.m.s. deviation = 0.030 Å) with the exception of the terminal methyl group which lies out of the plane [1.219 (3) Å]. The conformation about the C=N double bond [1.268 (3) Å] is *E*. An intra­molecular N—H⋯N hydrogen bond occurs. Linear supra­molecular chains along the *b* axis mediated by O—H⋯O hydrogen-bonding inter­actions feature in the crystal structure. These chains are also stabilized by weak C—H⋯N contacts.

## Related literature

For the preparation of isonitro­soacetanilides from aniline derivatives, see: Garden *et al.* (1997 ▶). For the use of isonitro­soacetanilides as precursors of pharmacologically important heterocyclic compounds, see: da Silva *et al.* (2001 ▶); Garden *et al.* (2002 ▶); Matheus *et al.* (2007 ▶); Maronas *et al.* (2008 ▶). For related structures, see: Briansó *et al.* (1974 ▶); Plana *et al.* (1976 ▶).
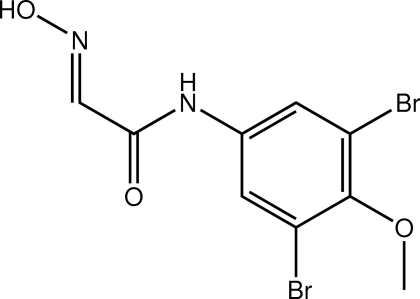


## Experimental

### 

#### Crystal data

C_9_H_8_Br_2_N_2_O_3_
*M* *_r_* = 351.98
Monoclinic, 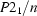

*a* = 10.3841 (2) Å
*b* = 8.8535 (1) Å
*c* = 13.0164 (3) Å
β = 106.356 (1)°
*V* = 1148.24 (4) Å^3^
*Z* = 4
Mo *K*α radiation
μ = 7.05 mm^−1^
*T* = 120 K
0.20 × 0.10 × 0.01 mm

#### Data collection

Nonius KappaCCD area-detector diffractometer
Absorption correction: multi-scan (*SADABS*; Sheldrick, 2007 ▶) *T*_min_ = 0.715, *T*_max_ = 1.000
14309 measured reflections
2643 independent reflections
2306 reflections with *I* > 2σ(*I*)
*R*_int_ = 0.036

#### Refinement

*R*[*F* ^2^ > 2σ(*F* ^2^)] = 0.028
*wR*(*F* ^2^) = 0.090
*S* = 1.16
2643 reflections
149 parameters
1 restraint
H-atom parameters constrained
Δρ_max_ = 0.77 e Å^−3^
Δρ_min_ = −0.86 e Å^−3^

### 

Data collection: *COLLECT* (Hooft, 1998 ▶); cell refinement: *DENZO* (Otwinowski & Minor, 1997 ▶) and *COLLECT*; data reduction: *DENZO* and *COLLECT*; program(s) used to solve structure: *SHELXS97* (Sheldrick, 2008 ▶); program(s) used to refine structure: *SHELXL97* (Sheldrick, 2008 ▶); molecular graphics: *ORTEP-3* (Farrugia, 1997 ▶) and *DIAMOND* (Brandenburg, 2006 ▶); software used to prepare material for publication: *publCIF* (Westrip, 2010 ▶).

## Supplementary Material

Crystal structure: contains datablocks global, I. DOI: 10.1107/S1600536810018623/hg2689sup1.cif

Structure factors: contains datablocks I. DOI: 10.1107/S1600536810018623/hg2689Isup2.hkl

Additional supplementary materials:  crystallographic information; 3D view; checkCIF report

## Figures and Tables

**Table 1:** Hydrogen-bond geometry (Å, °)

*D*—H⋯*A*	*D*—H	H⋯*A*	*D*⋯*A*	*D*—H⋯*A*
N1—H1n⋯N2	0.88	2.30	2.702 (3)	108
O2—H2o⋯O1^i^	0.84	1.83	2.672 (3)	175
C2—H2⋯N2^ii^	0.95	2.51	3.345 (3)	146

Symmetry codes: (i) 
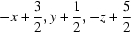
; (ii) 
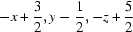
.

**Fig. 1.**
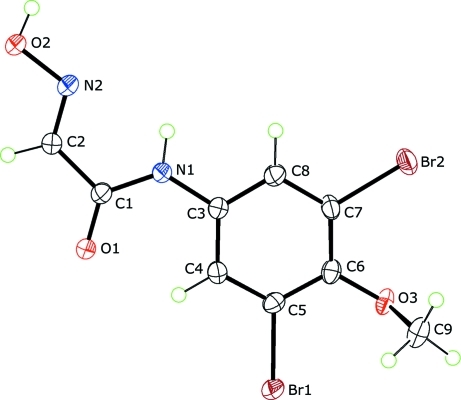
The molecular structure of (I) showing the atom-labelling scheme and displacement ellipsoids at the 50% probability level.

**Fig. 2.**
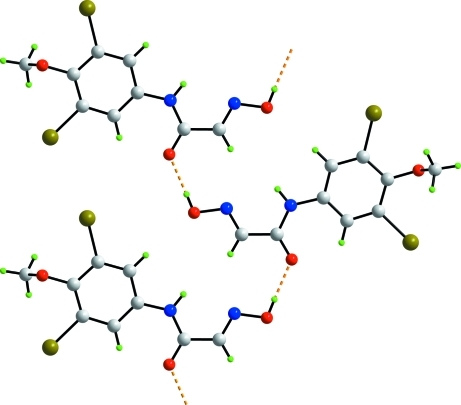
A view of a supramolecular array in (I) aligned along the b axis. The O–H···O hydrogen bonding interactions are shown as orange dashed lines. Colour code: Br, olive; O, red; N, blue; C, grey; and H, green.

## supplementary crystallographic information

### Comment

Isonitrosoacetanilides, readily available from aniline derivatives (Garden *et al.*, 1997), have found use as precursors of pharmacologically important heterocyclic compounds (da Silva *et al.*, 2001; Garden *et al.*, 2002; Matheus *et al.*, 2007; Maronas *et al.*, 2008).

The molecular structure of (I), Fig. 1, is essentially planar with the exception of the terminal methyl group. Thus, the r.m.s. deviation of all non-hydrogen atoms, excluding the methyl-C9 atom, is 0.030 Å; the C9 atom lies 1.219 (3) Å out of the plane. The conformation about the C2═N2 double bond [1.268 (3) Å] is *E*. The observed planarity is partially stabilised by an intramolecular N–H···N hydrogen bond (Table 1). There two other methoxy substituted 2-(hydroxyimino)-*N*-arylacetamide structures available for comparison,*i.e*. *o*-OMe (Plana *et al.*, 1976) and *p*-OMe (Briansó *et al.*, 1974) derivatives. The geometric parameters in these match closely those in (I). The major difference in the three structures relate to the non-planarity of (I) compared to the planarity in the literature structures. The proximity of the OMe group to two bromido substituents in (I) is the likely explanation for the deviation from planarity in (I). The crystal packing is dominated by O–H···O hydrogen bonding interactions that lead to the formation of a supramolecular linear chain along the *b* axis, Fig. 2 and Table 1. These chains are also stabilised by weak C–H···N contacts, Table 1.

### Experimental

The compound was prepared as previously reported from 3,5-dibromo-4-methoxyaniline, hydroxylamine.hydrogen sulfate in aqueous ethanol, containing sodium sulfate and CCl_3_CH(OH)_2_ (Garden *et al.*, 1997). The sample for the crystallographic study was recrystallised from EtOH, m.p. 463 K.

### Refinement

The O-, N- and C-bound H atoms were geometrically placed (O–H = 0.84 Å, N–H = 0.88 Å and C–H = 0.95–0.98 Å) and refined as riding with *U_iso_*(H) = 1.2-1.5*U_eq_*(parent atom).

### Crystal data

**Table:**

C_9_H_8_Br_2_N_2_O_3_	*F*(000) = 680
*M**_r_* = 351.98	*D*_x_ = 2.036 Mg m^−^^3^
Monoclinic, *P*2_1_/*n*	Mo *K*α radiation, λ = 0.71073 Å
Hall symbol: -P 2yn	Cell parameters from 2760 reflections
*a* = 10.3841 (2) Å	θ = 2.9–27.5°
*b* = 8.8535 (1) Å	µ = 7.05 mm^−^^1^
*c* = 13.0164 (3) Å	*T* = 120 K
β = 106.356 (1)°	Plate, colourless
*V* = 1148.24 (4) Å^3^	0.20 × 0.10 × 0.01 mm
*Z* = 4	

### Data collection

**Table:**

Nonius KappaCCD area-detector diffractometer	2643 independent reflections
Radiation source: Enraf Nonius FR591 rotating anode	2306 reflections with *I* > 2σ(*I*)
10 cm confocal mirrors	*R*_int_ = 0.036
Detector resolution: 9.091 pixels mm^-1^	θ_max_ = 27.5°, θ_min_ = 3.0°
φ and ω scans	*h* = −13→13
Absorption correction: multi-scan (*SADABS*; Sheldrick, 2007)	*k* = −11→10
*T*_min_ = 0.715, *T*_max_ = 1.000	*l* = −16→16
14309 measured reflections	

### Refinement

**Table:**

Refinement on *F*^2^	Primary atom site location: structure-invariant direct methods
Least-squares matrix: full	Secondary atom site location: difference Fourier map
*R*[*F*^2^ > 2σ(*F*^2^)] = 0.028	Hydrogen site location: inferred from neighbouring sites
*wR*(*F*^2^) = 0.090	H-atom parameters constrained
*S* = 1.16	*w* = 1/[σ^2^(*F*_o_^2^) + (0.0554*P*)^2^ + 0.0437*P*] where *P* = (*F*_o_^2^ + 2*F*_c_^2^)/3
2643 reflections	(Δ/σ)_max_ = 0.001
149 parameters	Δρ_max_ = 0.77 e Å^−^^3^
1 restraint	Δρ_min_ = −0.86 e Å^−^^3^

### Special details

**Table:**

Geometry. All s.u.'s (except the s.u. in the dihedral angle between two l.s. planes) are estimated using the full covariance matrix. The cell s.u.'s are taken into account individually in the estimation of s.u.'s in distances, angles and torsion angles; correlations between s.u.'s in cell parameters are only used when they are defined by crystal symmetry. An approximate (isotropic) treatment of cell s.u.'s is used for estimating s.u.'s involving l.s. planes.
Refinement. Refinement of *F*^2^ against ALL reflections. The weighted *R*-factor wR and goodness of fit *S* are based on *F*^2^, conventional *R*-factors *R* are based on *F*, with *F* set to zero for negative *F*^2^. The threshold expression of *F*^2^ > 2σ(*F*^2^) is used only for calculating *R*-factors(gt) etc. and is not relevant to the choice of reflections for refinement. *R*-factors based on *F*^2^ are statistically about twice as large as those based on *F*, and *R*- factors based on ALL data will be even larger.

### Fractional atomic coordinates and isotropic or equivalent isotropic displacement parameters (Å^2^)

**Table:**

	*x*	*y*	*z*	*U*_iso_*/*U*_eq_	
Br1	0.51024 (3)	0.51328 (3)	0.64286 (2)	0.03073 (12)	
Br2	0.57429 (3)	1.12760 (3)	0.76735 (2)	0.03212 (12)	
O1	0.6650 (2)	0.4317 (2)	1.04372 (14)	0.0253 (4)	
O2	0.7998 (2)	0.6540 (2)	1.37265 (15)	0.0268 (4)	
H2O	0.8150	0.7416	1.3979	0.040*	
O3	0.50326 (19)	0.8555 (2)	0.61613 (15)	0.0248 (4)	
N2	0.7577 (2)	0.6746 (2)	1.26323 (17)	0.0228 (5)	
N1	0.6742 (2)	0.6890 (2)	1.04718 (16)	0.0234 (5)	
H1N	0.6916	0.7664	1.0914	0.028*	
C1	0.6888 (3)	0.5517 (3)	1.0935 (2)	0.0216 (5)	
C2	0.7347 (3)	0.5508 (3)	1.2123 (2)	0.0235 (5)	
H2	0.7469	0.4577	1.2501	0.028*	
C3	0.6344 (3)	0.7255 (3)	0.9367 (2)	0.0217 (5)	
C4	0.5979 (3)	0.6159 (3)	0.8574 (2)	0.0221 (5)	
H4	0.5995	0.5118	0.8755	0.027*	
C5	0.5590 (3)	0.6618 (3)	0.7514 (2)	0.0218 (5)	
C6	0.5517 (2)	0.8133 (3)	0.7211 (2)	0.0221 (5)	
C7	0.5887 (3)	0.9197 (3)	0.8032 (2)	0.0238 (5)	
C8	0.6305 (3)	0.8778 (3)	0.9101 (2)	0.0242 (6)	
H8	0.6562	0.9524	0.9645	0.029*	
C9	0.6056 (3)	0.8606 (4)	0.5605 (2)	0.0332 (6)	
H9A	0.6683	0.9431	0.5894	0.050*	
H9B	0.5635	0.8776	0.4840	0.050*	
H9C	0.6544	0.7645	0.5704	0.050*	

### Atomic displacement parameters (Å^2^)

**Table:**

	*U*^11^	*U*^22^	*U*^33^	*U*^12^	*U*^13^	*U*^23^
Br1	0.0466 (2)	0.02593 (18)	0.01798 (18)	−0.00608 (11)	0.00633 (13)	−0.00244 (9)
Br2	0.0420 (2)	0.01905 (17)	0.0298 (2)	−0.00089 (10)	0.00100 (13)	0.00607 (10)
O1	0.0388 (11)	0.0189 (9)	0.0175 (9)	−0.0002 (8)	0.0067 (8)	0.0009 (7)
O2	0.0400 (11)	0.0222 (9)	0.0154 (9)	−0.0022 (8)	0.0033 (8)	0.0001 (7)
O3	0.0255 (9)	0.0305 (10)	0.0166 (9)	−0.0010 (7)	0.0031 (7)	0.0081 (7)
N2	0.0274 (12)	0.0235 (11)	0.0159 (11)	−0.0009 (8)	0.0033 (8)	0.0000 (8)
N1	0.0321 (12)	0.0184 (10)	0.0165 (11)	−0.0005 (9)	0.0015 (9)	0.0004 (8)
C1	0.0235 (12)	0.0220 (12)	0.0181 (13)	−0.0011 (10)	0.0042 (10)	0.0000 (10)
C2	0.0307 (14)	0.0196 (12)	0.0193 (13)	−0.0001 (10)	0.0054 (10)	0.0011 (10)
C3	0.0243 (12)	0.0209 (12)	0.0190 (13)	0.0013 (10)	0.0046 (10)	0.0015 (9)
C4	0.0246 (13)	0.0197 (12)	0.0215 (14)	0.0008 (9)	0.0056 (10)	0.0028 (9)
C5	0.0214 (12)	0.0250 (12)	0.0184 (13)	−0.0019 (10)	0.0045 (10)	−0.0018 (10)
C6	0.0188 (12)	0.0261 (13)	0.0195 (13)	0.0009 (10)	0.0022 (10)	0.0053 (10)
C7	0.0255 (13)	0.0184 (12)	0.0248 (14)	−0.0010 (10)	0.0028 (10)	0.0060 (10)
C8	0.0267 (13)	0.0244 (13)	0.0189 (13)	−0.0024 (10)	0.0024 (10)	0.0001 (9)
C9	0.0345 (16)	0.0420 (16)	0.0244 (15)	0.0030 (12)	0.0103 (12)	0.0102 (12)

### Geometric parameters (Å, °)

**Table:**

Br1—C5	1.892 (3)	C2—H2	0.9500
Br2—C7	1.894 (3)	C3—C4	1.390 (4)
O1—C1	1.233 (3)	C3—C8	1.390 (4)
O2—N2	1.379 (3)	C4—C5	1.385 (3)
O2—H2o	0.8400	C4—H4	0.9500
O3—C6	1.368 (3)	C5—C6	1.394 (4)
O3—C9	1.445 (3)	C6—C7	1.395 (4)
N2—C2	1.268 (3)	C7—C8	1.386 (4)
N1—C1	1.346 (3)	C8—H8	0.9500
N1—C3	1.417 (3)	C9—H9A	0.9800
N1—H1N	0.8800	C9—H9B	0.9800
C1—C2	1.484 (4)	C9—H9C	0.9800
			
N2—O2—H2o	104.6	C4—C5—C6	122.8 (2)
C6—O3—C9	113.1 (2)	C4—C5—Br1	118.79 (19)
C2—N2—O2	112.6 (2)	C6—C5—Br1	118.39 (19)
C1—N1—C3	128.6 (2)	O3—C6—C5	121.4 (2)
C1—N1—H1N	115.7	O3—C6—C7	121.7 (2)
C3—N1—H1N	115.7	C5—C6—C7	116.8 (2)
O1—C1—N1	124.3 (2)	C8—C7—C6	121.9 (2)
O1—C1—C2	120.1 (2)	C8—C7—Br2	119.2 (2)
N1—C1—C2	115.7 (2)	C6—C7—Br2	118.81 (19)
N2—C2—C1	119.9 (2)	C7—C8—C3	119.3 (2)
N2—C2—H2	120.0	C7—C8—H8	120.4
C1—C2—H2	120.0	C3—C8—H8	120.4
C4—C3—C8	120.6 (2)	O3—C9—H9A	109.5
C4—C3—N1	122.4 (2)	O3—C9—H9B	109.5
C8—C3—N1	117.0 (2)	H9A—C9—H9B	109.5
C5—C4—C3	118.5 (2)	O3—C9—H9C	109.5
C5—C4—H4	120.7	H9A—C9—H9C	109.5
C3—C4—H4	120.7	H9B—C9—H9C	109.5
			
C3—N1—C1—O1	−2.1 (4)	C4—C5—C6—O3	−175.0 (2)
C3—N1—C1—C2	178.9 (2)	Br1—C5—C6—O3	3.8 (3)
O2—N2—C2—C1	−179.9 (2)	C4—C5—C6—C7	1.3 (4)
O1—C1—C2—N2	−179.2 (3)	Br1—C5—C6—C7	−179.86 (19)
N1—C1—C2—N2	−0.2 (4)	O3—C6—C7—C8	176.2 (2)
C1—N1—C3—C4	2.9 (4)	C5—C6—C7—C8	−0.1 (4)
C1—N1—C3—C8	−178.4 (3)	O3—C6—C7—Br2	−1.3 (4)
C8—C3—C4—C5	0.7 (4)	C5—C6—C7—Br2	−177.56 (18)
N1—C3—C4—C5	179.3 (2)	C6—C7—C8—C3	−0.8 (4)
C3—C4—C5—C6	−1.7 (4)	Br2—C7—C8—C3	176.7 (2)
C3—C4—C5—Br1	179.57 (19)	C4—C3—C8—C7	0.5 (4)
C9—O3—C6—C5	−88.4 (3)	N1—C3—C8—C7	−178.2 (2)
C9—O3—C6—C7	95.4 (3)		

### Hydrogen-bond geometry (Å, °)

**Table:**

*D*—H···*A*	*D*—H	H···*A*	*D*···*A*	*D*—H···*A*
N1—H1n···N2	0.88	2.30	2.702 (3)	108
O2—H2o···O1^i^	0.84	1.83	2.672 (3)	175
C2—H2···N2^ii^	0.95	2.51	3.345 (3)	146

Symmetry codes:  (i) −*x*+3/2, *y*+1/2, −*z*+5/2; (ii) −*x*+3/2, *y*−1/2, −*z*+5/2.
